# Can the Gene Expression Profile of Patients with Acute Myeloid Leukemia Predict Complete Remission Following Induction Therapy?

**DOI:** 10.7150/jca.57457

**Published:** 2022-02-14

**Authors:** Santiago Sánchez-Sosa, Carlos Rodríguez-Medina, Ruth Stuckey, Yanira Florido, Guillermo Santana, Jesús María González Martín, Naylén Cruz-Cruz, Melissa Torres-Ochando, Rosa Fernández, Nuria Sánchez-Farías, Antonia Cionfrini, Teresa Molero Labarta, María Teresa Gomez-Casares, Cristina Bilbao-Sieyro

**Affiliations:** 1 Hematology Department, Hospital Universitario de Gran Canaria Dr. Negrín, Las Palmas de Gran Canaria, Spain.; 2 Medical Science Department, Universidad de Las Palmas de Gran Canaria, Las Palmas de Gran Canaria, Spain.; 3 Unidad de Investigación, Hospital Universitario de Gran Canaria Dr. Negrín, Las Palmas de Gran Canaria, Spain.; 4 Hematology Department, Complejo Hospitalario Universitario Insular Materno Infantil, Las Palmas de Gran Canaria, Spain.; 5 Morphology Department, Universidad de Las Palmas de Gran Canaria, Las Palmas de Gran Canaria, Spain.

**Keywords:** acute myeloid leukemia, biomarkers, patient outcome, induction therapy, molecular diagnostics

## Abstract

Recent advances in sequencing technologies and genomics have led to the development of several targeted therapies such as BCL2 and Bromodomain and extra-terminal (BET) protein inhibitors for a more personalized treatment of patients with acute myeloid leukemia (AML), yet the majority of patients still receive standard induction chemotherapy. The molecular profiles of patients who are likely to respond to induction therapy and novel directed therapies remain to be determined. The expression of AML-related genes that are targeted by novel therapies such as *BCL2* and *BRD4*, as well as functionally related genes and associated epigenetic modulators (*TET2*,* EZH2*,* ASXL1*,* MYC*) were analyzed in a series of 176 consecutive AML patients at multiple points during the disease course - diagnosis (Dx), post-induction (PI), complete remission (CR) and relapse (RL) - and their relationship with clinical variables and outcome investigated. Higher *TET2* expression was observed PI and at CR compared to Dx, with significantly superior *TET2* expression after induction therapy in the group of patients who reached CR compared to those who did not. Thus, the upregulation of *TET2* at PI may be a marker of CR in AML patients. On the other hand, cells with high levels of *MYC* and *BCL2* may be vulnerable to BRD4 inhibition.

## Introduction

Acute myeloid leukemia (AML) is one of the most common leukemias, accounting for 30% of adult cases, with a median age of presentation of 68 years 1. Although AML is a highly heterogenous disease, the vast majority of patients still receive standard induction chemotherapy (anthracycline + cytarabine, “7+3”). However, approximately 65%-70% of patients aged 60 and under 2 and just 30%-60% of the over-60s obtain complete remission (CR) 3, 4, with failure to achieve CR associated with a very poor outcome 5. As a result, current outcomes for AML patients remain unsatisfactory: those aged 60 years and under have a long-term disease-free survival (DFS) of only 40%, whereas the DFS of older patients is 10% or less, with a median overall survival of less than one year, regardless of therapeutic approach 6, 7.

Recent progress in the use of massive sequencing technologies has led to a greater understanding of the disease pathology. This knowledge has in turn fueled the development of targeted therapies, such as those developed against *FLT3* and *IDH1/2* mutations 8. BCL2 inhibitors (venetoclax was approved by the FDA on November 21, 2018, in combination with azacitidine or decitabine or low-dose cytarabine for the treatment of newly-diagnosed AML in adults aged 75 years or older 9) and Bromodomain and extra-terminal (BET) protein inhibitors (showing promising results in recent clinical trials 10), both of which induce apoptosis, are also offering hope for AML patients.

Nevertheless, the molecular profiles of patients who are likely to respond to these novel targeted therapies remain to be determined. Moreover, algorithms to predict which patients are unlikely to respond well to the standard induction regime, and thus could benefit from an alternative therapy, do not exist and represent an unmet clinical need. Therefore, there is still much work to be done in personalizing the risk stratification and treatment regimes of AML patients in the clinic.

In this study, we determined the expression of AML-related genes that are targeted by novel therapies in AML such as *BCL2* and *BRD4*, as well as functionally related genes and associated epigenetic modulators (*TET2*,* EZH2*,* ASXL1*,* MYC*) in a series of patients with AML at diagnosis and at different points during follow-up and evaluated whether their expression was predictive of complete remission after induction and/or patient outcome.

## Materials & Methods

In this study, approved by our center's IRB (Comité Ético de Investigación Clínica, CEI_HUGCDN_565/150024), we analyzed 176 consecutive AML patients diagnosed at the Hospital Universitario de Gran Canaria Dr. Negrín and the Complejo Hospitalario Universitario Materno Infantil, Las Palmas, Spain, from January 2014 to July 2017.

Bone marrow (BM) and peripheral blood (PB) samples were collected at diagnosis (Dx), post-induction (PI, which corresponded to the BM evaluation at day 21 after induction therapy), complete remission (CR, defined as <5% blasts in bone marrow, absence of extramedullary disease, neutrophils ≥1.0 × 10^9^/L and platelets ≥100 × 10^9^/L, according to the 2017 ELN recommendations 11) and at relapse (RL).

RNA was extracted from bone marrow cell pellets using the Qiacube automatic extractor (Qiagen) and cDNA synthesized with random hexamers (Roche) and M-MLV Reverse Transcriptase (Promega). Real-time quantitative PCR (RT-qPCR) was conducted with Perfecta SYBR Green FastMix (Quanta Bioscience) on the LightCycler 480 platform (Roche), with *ABL1* as reference gene.

Gene expression was determined by the 2-∆∆Ct method normalized to *ABL1* and relative to a cDNA pool from 10 healthy donors as internal calibrator; *MYC* expression was normalized to *RPS14*. Primer sequences are available upon request. Expression of targets genes was analyzed separately in the healthy donors with no significant variation observed.

Pearson correlation coefficients were used to calculate correlations between two continuous variables. For the comparison between continuous variables, paired Student's t-test for parametric data or Wilcoxon signed-rank test for non-parametric variables was used. Univariate and multivariate survival analyses were carried out using the Cox proportional hazard model only for patients who received first-line intensive treatment (anthracycline + cytarabine, 7 + 3 schedule). Progression-free survival was defined as the time from diagnosis to disease progression or death from any cause (PFS). All statistical analyses were two-sided, with statistical significance was set at a p-value < 0.05. Analyses were carried out using the R Core Team 2019 (version 3.6.1).

Informed consent was provided by all patients and donors. The datasets from this study are available from the corresponding author on reasonable request.

## Results

### Patient characteristics

Median patient age was 59 years (min-max: 16-82 years, see Supplementary Table 1 for patient characteristics). A *de novo* AML was diagnosed in 150 patients, while 26 were secondary AML (therapy-related or with an antecedent hematologic disorder). The majority of patients (94.3%) received standard induction chemotherapy (anthracycline + cytarabine, “7+3”), and 38.6% of patients received hematopoietic stem cell transplantation in consolidation. Of the patients who received induction chemotherapy, 89 (50.6%) reached CR.

### Gene expression correlation

**Figure 1:**
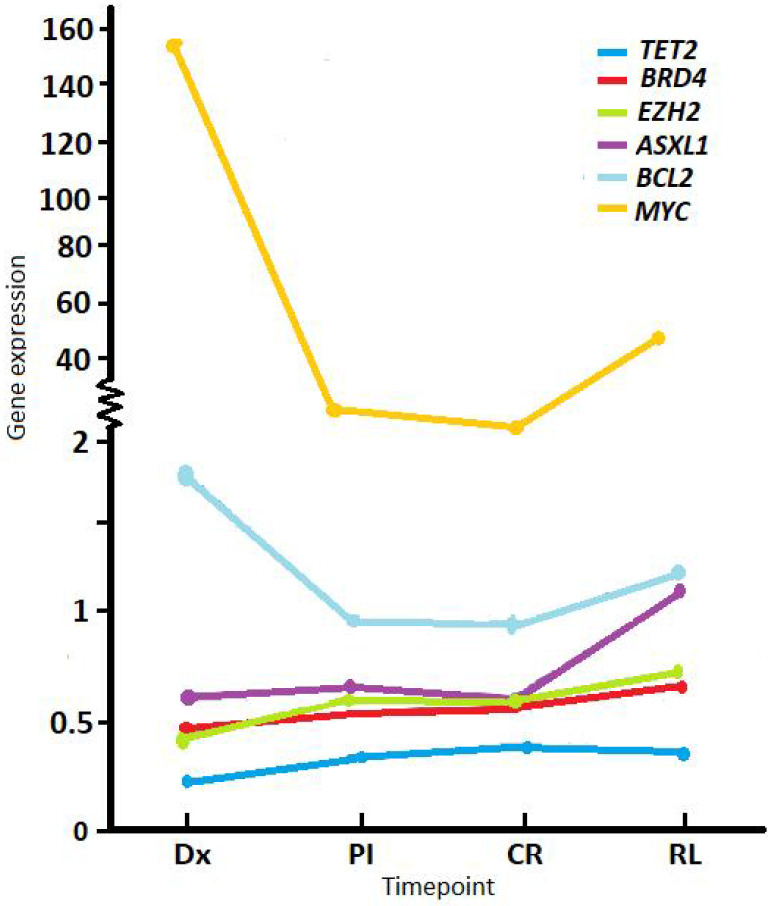
** Average expression levels of study genes at different timepoints during follow up.** Normalized expression shown at diagnosis (Dx), post-induction (PI), complete remission (CR) and relapse (RL). Values were normalized to the *ABL1* reference gene and relative to a pool of healthy donors.

RT-qPCR was performed on RNA extracted from whole BM for patients with a sample available that was suitable for RNA expression analysis (e.g., not degraded). Mean gene expression levels at diagnosis (normalized to *ABL1* and relative to a pool of healthy donor controls) were below the values of controls for *TET2* (mean 0.22, SD ± 0.15, min-max 0-0.91, n = 153), *EZH2* (mean 0.43, SD ± 0.29, min-max 0.04-1.73, n = 155), *BRD4* (mean 0.47, SD ± 0.37, min-max 0.05-2.59, n = 158), and *ASXL1* (mean 0.66, SD ± 0.44, min-max 0.08-2.85, n = 155); and were higher compared to controls for *BCL2* (mean 1.61, SD ± 1.16, min-max 0.02-6.97, n = 156) and *MYC* (mean 151.27, SD ± 346.23, min-max 0.1-1929, n = 157) (Figure 1).

**Figure 2:**
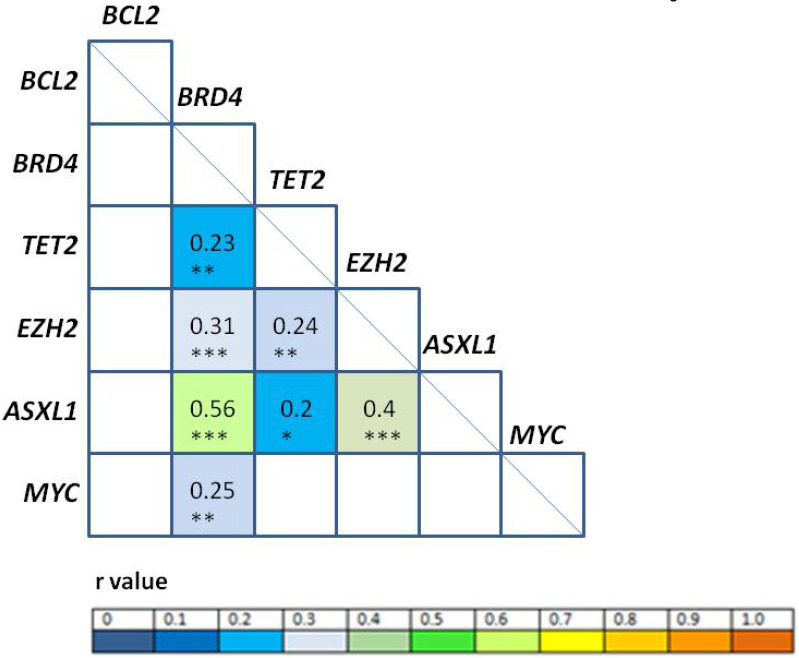
** Correlation analysis of gene expression levels.** The r values (Pearson correlation coefficient) of statistically significant correlations are represented according to the color heat map; white squares were not significant. *p<0.05, **p<0.01, ***p<0.001.

Correlation analyses among gene expression levels at diagnosis (Figure 2) revealed positive correlation between *BRD4/ASXL1* (*r* = 0.56, p < 0.001), *EZH2/ASXL1* (*r* = 0.4, p < 0.001); *BRD4/EZH2* (*r* = 0.31, p < 0.001), *BRD4/MYC* (*r* = 0.25, p = 0.01), *TET2/EZH2* (*r* = 0.24, p = 0.01), *BRD4/TET2* (*r* = 0.23, p = 0.01), and *TET2*/*ASXL1* (*r* = 0.2; p = 0.03). High correlation (Pearson correlation coefficient r ≥ 0.8) was also observed between *EZH2/ASXL1* at PI (*r* = 0.82, p < 0.001), CR (*r* = 0.92, p < 0.001); and at relapse (*r* = 0.85, p < 0.001).

### Expression levels during follow-up

Comparing expression levels at Dx with PI, *TET2* showed a significant increase (mean Dx 0.22 *vs.* PI 0.33, p < 0.001), while *BCL2* (mean Dx 1.64 *vs*. PI 1.18, p < 0.001) and *MYC* (mean Dx 151.27 *vs.* PI 2.82, p < 0.001) a marked descent PI. We observed the same behavior between Dx and CR (Table 1). Between Dx and RL, *MYC* levels were significantly lower at RL (mean Dx 67.22 *vs*. RL 49.13, p = 0.01).

When expression at either PI or CR was compared with RL, the only gene with significantly modified expression values was *BCL2,* which showed a significant increment at RL (mean PI 1.07 and CR 0.96 *vs*. RL 2.17, p = 0.05 and p = 0.03, respectively) 12.

**Figure 3:**
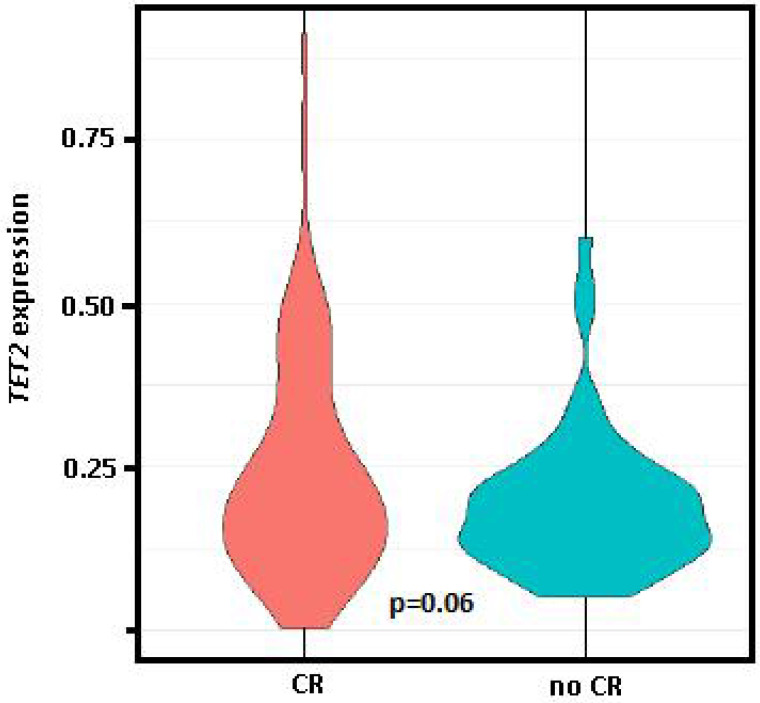
** Violin plot of *TET2* expression at diagnosis.** Comparison of the group who achieved complete remission (CR) after induction therapy *vs.* those with persistence and/or exitus (no CR). Student t test (parametric) used for distinct distributions, p=0.06.

Comparing the gene expression levels of patients at PI who had obtained CR in response to induction therapy (n = 89, 50.6%) *vs.* those who had persistence and/or exitus (n = 77, 43.8%), only *TET2* had significantly different expression levels between the two groups (0.37 *vs.* 0.26, p = 0.03), while differences between these groups for *TET2* expression levels at diagnosis was of marginal significance (0.24 *vs.* 0.19, p = 0.06, Figure 3).

### Clinical variables

In terms of clinical variables, no association was found between expression of the genes analyzed at diagnosis with patient age, or leukocyte, hemoglobin, creatinine or lactate dehydrogenase (LDH) levels. However, a significant inverse correlation was observed between blasts in PB and expression of *BRD4* (r = -0.26, p= 0.01)*, EZH2* (r = -0.27, p = 0.01) and *ASXL1* (r = -0.2, p = 0.04) at diagnosis (Supplementary Table 2).

When included in a predictive model, a 1-fold increase in *EZH2* and *BRD4* expression caused a 21.05-fold (p = 0.04) and 13.36-fold (p = 0.1) decrease in blasts in PB, respectively; whereas a 1-fold increase in *EZH2* and *ASXL1* expression caused a 13.81-fold decrease (p = 0.1) and a 11.27-fold increase (p = 0.05) in blasts in BM (Supplementary Figure 1).

### Overall and progression-free survival

Median survival in our series was 14.8 months, with 57.4% of patients alive after 12 months. As expected, age at diagnosis was predictive of overall survival (OS) for the whole series (p < 0.001, Table 2) 13, but also for patients aged 70 years and under with intermediate cytogenetic risk (p = 0.037).

Univariate analysis for OS revealed no association between gene expression levels at diagnosis or at relapse (Table 2). Moreover, Cox regression analysis revealed no association between gene expression at diagnosis and the time between diagnosis and exitus.

At PI, the expression levels of *ASXL1* (p = 0.034) and *BCL2* (p = 0.046) were associated with OS, with *MYC* expression of marginal significance (p = 0.062); while the levels of *ASXL1* (p < 0.001), *BCL2* (p = 0.012), *MYC* (p = 0.041) and* EZH2* (p < 0.001) were associated with OS at CR, with *BRD4* expression of marginal significance (0.48 in group of survivors *vs.* 0.66 in exitus group, p = 0.062). In the multivariate analysis, age and leukocyte count at diagnosis, ELN risk group 3, and *BCL2* expression at both PI and CR retained significance 12.

Median PFS in our series was 12.5 months. Univariate analysis revealed no association between gene expression levels at diagnosis or at relapse. However, the gene expression levels of *ASXL1* and *BCL2* at PI (albeit of marginal significance, p = 0.073 and 0.06, respectively) and of *ASXL1, BCL2* and *EZH2* at CR (p = 0.016, 0.022, 0.019, respectively) were associated with PFS (Table 3).

## Discussion

In accordance with a previous report, we observed significantly higher *TET2* expression after induction and at complete remission compared to diagnosis, although we did not observe a reduction in *TET2* expression at relapse 14. Importantly, *TET2* expression was lower at PI in those patients who achieved CR in response to induction therapy compared to those who did not. Meanwhile, *BCL2* and *MYC* levels were significantly lower after induction and at CR compared to diagnosis.

In this expression study, *BCL2* was the only gene to show a significant increase in expression at relapse compared to PI and CR 12. Early studies showed that overexpression of *BCL2* is a common event in AML and that high levels are associated with chemoresistance and low complete remission rate 15-17. Further papers demonstrated that it is the readiness for apoptosis of myeloblasts, known as “priming”, rather than the monogenic expression of a single apoptosis-related gene at diagnosis (i.e. *BAX*, *BLC2*), that determines a successful response to chemotherapy 18, 19.

Relapsed AML cases have a particularly poor prognosis due to limited treatment options for refractory cases. As the sensitivity to the selective BCL-2 inhibitor ABT-199 seems to correlate with *BCL2* levels 20, it is possible that *BCL2* expression at PI, at CR and even at relapse, may determine candidates for venetoclax as an alternative treatment.

Our study is the first to analyze *BRD4* expression in a series of patients with AML at different timepoints during patient follow up, namely, after induction therapy, when the patients reached CR, and at relapse. No significant associations were observed for *BRD4* expression during patient follow up, although expression levels at CR were marginally associated with OS. This result is in accordance with previous results associating high BRD4 expression with poor OS in response to chemotherapy 21.

Although no association was observed between gene expression levels at diagnosis and OS or PFS, expression levels of *ASXL1* and *BCL2* at PI and of *EZH2, ASXL1, BCL2* and *MYC* at CR were associated with OS, while *EZH2, ASXL1* and *BCL2* levels at CR were associated with PFS. Assessment of post-treatment remission is currently primarily based on cytomorphology, with CR defined as <5% blasts in the bone marrow 11, but this does not mean that the patient is free of disease. In fact, the consensus among experts is to evaluate for the presence of measurable residual disease (MRD), defined as post-therapy persistence of leukemic cells at levels below morphologic detection, since it is a strong, independent prognostic marker of increased risk of relapse and shorter survival 22. Our results suggest that gene expression at PI/CR has a significant influence on patient outcome. Therefore, expression analysis of a set of just six genes could help refine the risk stratification of patients who achieve CR in response to standard induction therapy to better predict patient survival and identify patients likely to require an alternative treatment approach. Unlike other multi-gene prediction scores (such as the 17- or 29-gene scores 23, 24), our RT-qPCR strategy is simple, cost-efficient and easily applicable in most hematology laboratories.

As expected, we observed that the expression levels at diagnosis of genes with a tumor suppressor function in AML (*TET2, EZH2* and *ASXL1*) were low compared to normal bone marrow, whereas levels at diagnosis of genes with an oncogenic role (*BCL2* and *MYC*) were higher in a series of 176 AML patients. *TET2* levels were the lowest with regard to normal bone marrow, in agreement with what has already been described in AML 14.

We observed a very positive correlation between *ASXL1* and *EZH2* expression at diagnosis, PI and relapse, which may correspond to their cooperative functions in relation to the H3K27me3 epigenetic mark 25. In addition, there was a correlation between *EZH2/ASXL1* and *TET2* levels at diagnosis that could be explained by the described collaborative role of the malfunction of ASXL1 and TET2 in promoting the commitment of hematopoietic cells to the myeloid lineage in myeloid pathogenesis 26. The association found between *BRD4* and *MYC* agrees with the biological activating function of BRD4 over *MYC* expression 27. Finally, *BRD4* expression also correlated with *EZH2/ASXL1/TET2;* accordingly, it has been published that BRD4 regulates *EZH2* transcription through the upregulation of *MYC* 27 and binds directly to enhancer sites in the *EZH2, MYC* and *BCL2* genes 28. Therefore, there is biological concordance with our results of observed mRNA expression levels at diagnosis. These correlations may also be important to take into account when considering combination therapies.

Moreover, although significant associations were observed between *BRD4* and *MYC* levels but not between *BRD4* and *BCL2* levels, the expression of *BCL2* and *MYC*, both downstream targets of BRD4 28, 29, were significantly lower after induction and at CR compared to diagnosis. Interestingly, studies have shown that the treatment of AML cells with the selective small-molecule bromodomain inhibitor JQ1 caused the rapid downregulation of *BCL2* and *MYC* transcription, followed by genome-wide downregulation of Myc-dependent target genes 27, 29. Therefore, cells with high levels of *MYC* and *BCL2* may be vulnerable to BRD4 inhibition. Moreover, combination treatment with BET inhibitors and venetoclax has recently been reported to be more effective in inducing lethal effects against AML blasts, without inducing toxicity, in AML engrafted mice 30.

In conclusion, gene expression at PI/CR has a significant influence on patient outcome. Our findings support the upregulation of *TET2* and the downregulation of *BCL2* and *MYC* at post-induction as potential follow-up targetable markers in AML. However, further analyses in a larger AML series are needed to confirm that expression analysis of a set of just six genes could help refine the risk stratification of patients who achieve complete remission in response to standard induction therapy and to establish which patients are likely to benefit from therapy with BCL2 and/or BRD4 inhibitors.

## Supplementary Material

Supplementary figure and tables.

## Figures and Tables

**Table 1:** ** Comparison of average gene expression levels at different timepoints during follow up.** Values were normalized to the *ABL1* reference gene and relative to a pool of healthy donors (expression level set as 1 for each gene)

	Nº	Dx	PI	CR	RL	p-value
*TET2*	82	**0.22**	**0.33**			**<0.001**
*BRD4*	86	0.47	0.54			0.204
*EZH2*	85	0.45	0.6			0.16
*ASXL1*	84	0.63	0.66			0.69
*BCL2*	86	**1.64**	**0.97**			**<0.001**
*MYC*	83	**151.27**	**2.82**			**<0.001**
*TET2*	60	**0.22**		**0.38**		**<0.001**
*BRD4*	64	0.48		0.58		0.18
*EZH2*	63	0.46		0.61		0.25
*ASXL1*	63	0.65		0.62		0.72
*BCL2*	64	**1.57**		**0.95**		**<0.001**
*MYC*	61	**135.5**		**2.4**		**0.002**
*TET2*	27	0.25			0.36	0.26
*BRD4*	27	0.52			0.67	0.39
*EZH2*	27	0.54			0.73	0.87
*ASXL1*	27	0.71			1.09	0.1
*BCL2*	28	1.65			1.19	0.77
*MYC*	27	**67.22**			**49.13**	**0.01**
*TET2*	65		0.36	0.38		0.1
*BRD4*	66		0.56	0.58		0.28
*EZH2*	66		0.65	0.61		0.07
*ASXL1*	66		0.65	0.63		0.26
*BCL2*	66		0.99	0.97		0.74
*MYC*	64		3.02	2.34		0.25
*TET2*	21		0.34		0.35	0.24
*BRD4*	22		0.64		0.73	0.78
*EZH2*	22		0.81		0.79	0.94
*ASXL1*	22		0.77		1.19	0.07
*BCL2*	23		**1.07**		**2.17**	**0.05**
*MYC*	21		1.98		56.62	0.22
*TET2*	18			0.39	0.36	0.18
*BRD4*	19			0.72	0.77	0.31
*EZH2*	19			0.88	0.86	0.78
*ASXL1*	19			0.81	1.27	0.2
*BCL2*	20			**0.96**	**2.27**	**0.03**
*MYC*	18			2.43	65.79	0.71

Significant differences are shown in **bold.** Nº: number of observations considered in each analysis; Dx: diagnosis; PI: post-induction; CR: complete remission; RL: relapse.

**Table 2:** ** Univariate and multivariate analysis for overall survival for the whole series.** Average expression levels for the group of survivors and non-survivors are included for genes whose expression at certain timepoints was significant (or of borderline significance)

Variables	Nº	HR	p-value	Average expr.	
				Survivors	Non-survivors
***Univariate***					
Male gender	176	1.03	0.87		
Age at diagnosis (continuous)	176	1.04	**<0.001**		
ELN risk group*	160				
2		1.53	0.12		
3		2.10	**0.008**		
Cytogenetic risk group*	173				
2		1.30	0.48		
3		2.07	0.07		
Leucocytes (log) at diagnosis	173	1.13	**0.05**		
**At diagnosis**	153				
*TET2* expr.		0.47	0.26		
*BRD4* expr.		0.72	0.19		
*EZH2* expr		0.73	0.37		
*ASXL1* expr.		0.87	0.54		
*BCL2* expr.		1.08	0.37		
*MYC* expr.		1	0.88		
**Post-induction**	90				
*TET2* expr.		0.39	0.15		
*BRD4* expr.		1.25	0.5		
*EZH2* expr.		1.26	0.089		
*ASXL1* expr.		1.63	**0.034**	0.57	0.72
*BCL2* expr.		1.45	**0.046**	0.81	1.07
*MYC* expr.		1.03	0.062	1.26	4.26
**Complete remission**	67				
*TET2* expr.		0.95	0.94		
*BRD4* expr		1.94	0.062	0.48	0.66
*EZH2* expr.		1.45	**<0.001**	0.45	0.76
*ASXL1* expr.		1.98	**<0.001**	0.52	0.72
*BCL2* expr.		1.83	**0.012**	0.81	1.10
*MYC* expr		1.08	**0.041**	1.31	3.27
**At relapse**	29				
*TET2* expr.		0.33	0.12		
*BRD4* expr.		1.05	0.87		
*EZH2* expr.		0.99	0.96		
*ASXL1* expr.		1.06	0.65		
*BCL2* expr.		1.11	0.12		
*MYC* expr.		1	0.23		
***Multivariate***					
Age at diagnosis	158	1.04	**<0.001**		
ELN risk group*	158				
2		1.40	0.233		
3		1.76	**0.044**		
Leucocytes (log) at diagnosis	158	1.24	**0.003**		
*BCL2* expr. PI	82	1.58	**0.014**	0.81	1.07
*BCL2* expr. CR	60	1.96	**0.008**	0.81	1.10

Significant differences are shown in **bold.** Nº: number of observations considered in each analysis; PI: post-induction; CR: complete remission; RL: relapse; ELN: European LeukemiaNet. * Risk group 1 taken as reference.

**Table 3:** ** Univariate analysis for progression-free survival for the whole series.** Significant differences are shown in bold

Variables	HR	p-value
Male gender	1.07	0.74
Age at diagnosis (continuous)	1.02	**<0.001**
ELN risk group*		
2	1.20	0.50
3	1.71	**0.04**
**At diagnosis**		
TET2 expr.	0.62	0.45
*BRD4* expr.	0.67	0.1
*EZH2* expr.	1.12	0.73
*ASXL1* expr.	0.72	0.16
*BCL2* expr.	1.09	0.3
*MYC* expr.	1	0.84
**Post-induction**		
*TET2* expr.	0.38	0.11
*BRD4* expr.	1.08	0.8
*EZH2* expr.	1.23	0.13
*ASXL1* expr.	1.52	0.073
*BCL2* expr.	1.39	0.06
*MYC* expr.	1.02	0.19
**Complete remission**		
*TET2* expr.	0.83	0.77
*BRD4* expr.	1.57	0.18
*EZH2* expr.	1.37	**0.019**
*ASXL1* expr.	1.8	**0.016**
*BCL2* expr.	1.63	**0.022**
*MYC* expr.	1.05	0.15
**At relapse**		
*TET2* expr.	0.88	0.83
*BRD4* expr.	1.02	0.95
*EZH2* expr.	1.1	0.63
*ASXL1* expr.	1.02	0.86
*BCL2* expr.	1.08	0.24
*MYC* expr.	1	0.39

*Risk group 1 taken as reference. ELN: European LeukemiaNet.
