# Supplementary material for: Can the Gene Expression Profile of Patients with Acute Myeloid Leukemia Predict Complete Remission Following Induction Therapy?
Source: J Cancer. 2022 Feb 14;13(4):1356–62. doi: 10.7150/jca.57457 (PMC8899374; doi:10.7150/jca.57457)

Supplementary Table 1. Characteristics of the study population.

Characteristics	N (%)
**Diagnosis**	
De novo AML	150 (85.2)
Secondary AML, including therapy-related AML	26 (14.8)
**Sex**	
Female	73 (41.5)
Male	103 (58.5)
**Median age at diagnosis** (years)(range)	59 (16-82)
**Blood counts at diagnosis:**	
**Leukocytes** per µL (mean)(range)	6900 (900–412000)
**Hemoglobin** g/dL (mean)(range)	8.8 (1.8–14.4)
**Platelets** per µL (mean)(range)	82474 (2200–1150000)
**Blasts in peripheral blood** (%)(range)	36 (0–100)
**Blasts in bone marrow** (%)(range)	57 (14–99)
**LDH** µ/L (mean)(range)	600 (97–3842)
**Cytogenetic risk**	
Low	13 (7.4)
Intermediate	122 (69.3)
High	38 (21.6)
Unknown	3 (1.7)
**ELN risk category**	
Low	37 (21.0)
Intermediate	66 (37.5)
High	57 (32.4)
Unknown	16 (9.1)
NPM1	
Wild type	128 (72.7)
Mutated	41 (23.3)
Unknown	7 (4)
***FLT3*-ITD**	
Wild type	139 (79)
Mutated	31 (17.6)
Unknown	6 (3.4)
**Treatment**	
Intensive induction therapy: anthracycline + cytarabine (7+3)	166 (94.3)
Other	9 (5.1)
Exitus prior to induction	1 (0.6)
**Response to induction** *	
Complete remission	89 (50.6)
Primary refractory disease	67 (38.1)

Did not receive induction	10 (5.7)
**Allogeneic HCT in consolidation ***	
No	92 (55.4)
Yes	74 (44.6)
**Relapse**	
No	119 (67.6)
Yes	57 (32.4)
**Survival indicators:**	
**Progression-free survival**, months (median)(range)	12.5 (0.0–147.0)
**Event-free survival**, months (median)(range)	13.3 (0.1–148.2)
**Survival**, months (median)(range)	14.8 (0.2–147.0)
**Alive at 1-year** (n)(%)	101 (57.4)
**Exitus**	117 (66.5)

N: number of observations considered in each analysis; AML: acute myeloid leukemia; ELN: European LeukemiaNet; HCT: hematopoietic cell transplantation.

* 10 patients died during induction

Supplementary Table 2. Correlation between gene expression and clinical variables at diagnosis.

Clinical variable	Gene	r	p-value
Age	-	-	ns
Leucocyte count	-	-	ns
Hemoglobin	-	-	ns
Creatinine	-	-	ns
LDH	-	-	ns
Platelets	*BCL2*<br>*ASXL1*	-0.16<br>0.16	0.06<br>0.08
Blasts in PB	*BRD4*<br>*EZH2*<br>*ASXL1*	-0.26<br>-0.27<br>-0.2	**0.01**<br>**0.01**<br>**0.04**

R: Pearson coefficient; ns, not significant; LDH: lactate dehydrogenase; PB: peripheral blood.

Supplementary Figure 1. Prediction of blasts in (A) peripheral blood (PB) according to *EZH2* and *BRD4* expression, and (B) bone marrow (BM) according to *EZH2* and *ASXL1* expression at diagnosis. Variables selected as predictors of blasts using the Genetic Algorithm model.

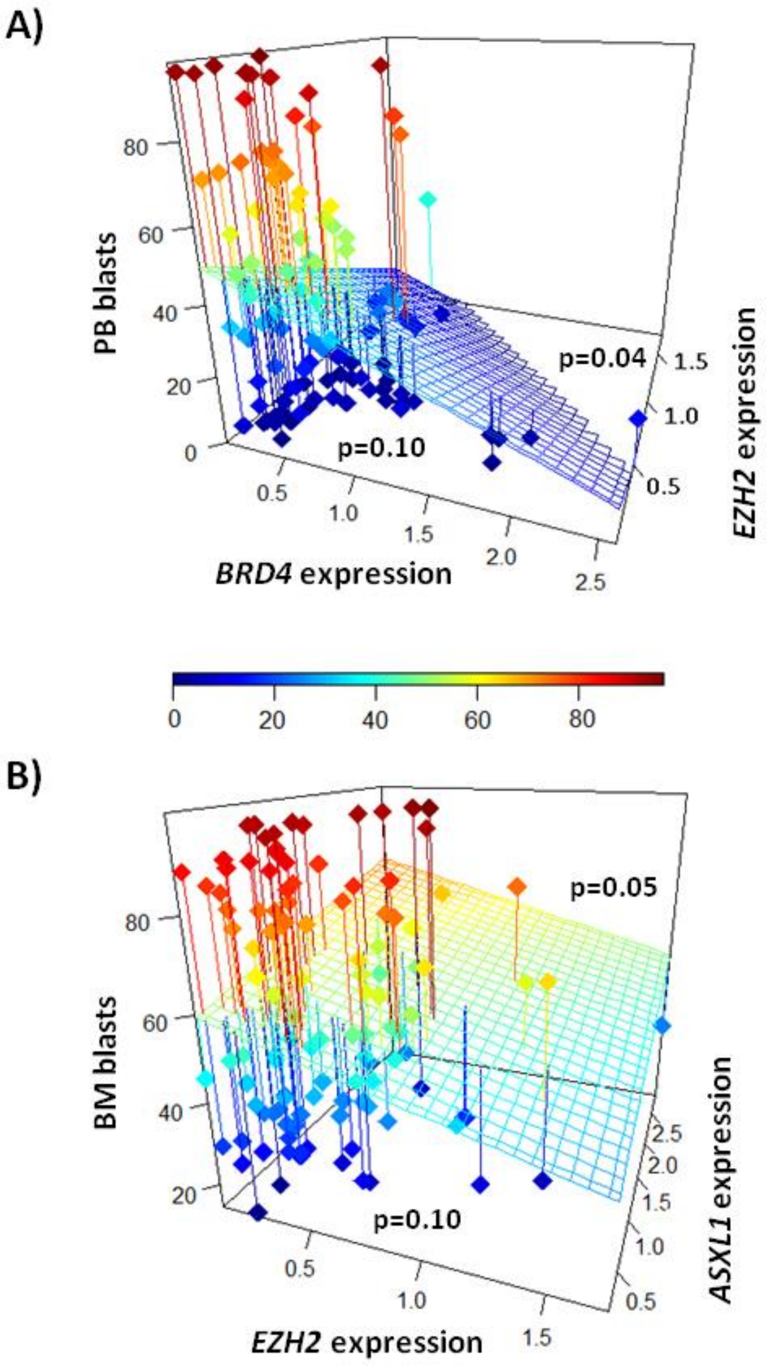

Supplement: Supplementary file 1 — Supplementary figure and tables. [file jcav13p1356s1.pdf]
